# Mitochondrial metabolism in *Drosophila* macrophage-like cells regulates body growth via modulation of cytokine and insulin signaling

**DOI:** 10.1242/bio.059968

**Published:** 2023-11-29

**Authors:** Shrivani Sriskanthadevan-Pirahas, Abdul Qadeer Tinwala, Michael J. Turingan, Shahoon Khan, Savraj S. Grewal

**Affiliations:** Clark H Smith Brain Tumour Centre, Arnie Charbonneau Cancer Institute, Alberta Children's Hospital Research Institute, and Department of Biochemistry and Molecular Biology Calgary, University of Calgary, Alberta T2N 4N1, Canada

**Keywords:** Mitochondria, Hemocytes, Systemic growth, Insulin signaling, Cytokine TNF-α/Eiger, JNK signaling, TFAM, OxPhos, Metabolism, *Drosophila*

## Abstract

Macrophages play critical roles in regulating and maintaining tissue and whole-body metabolism in normal and disease states. While the cell–cell signaling pathways that underlie these functions are becoming clear, less is known about how alterations in macrophage metabolism influence their roles as regulators of systemic physiology. Here, we investigate this by examining *Drosophila* macrophage-like cells called hemocytes. We used knockdown of TFAM, a mitochondrial genome transcription factor, to reduce mitochondrial OxPhos activity specifically in larval hemocytes. We find that this reduction in hemocyte OxPhos leads to a decrease in larval growth and body size. These effects are associated with a suppression of systemic insulin, the main endocrine stimulator of body growth. We also find that TFAM knockdown leads to decreased hemocyte JNK signaling and decreased expression of the TNF alpha homolog, Eiger in hemocytes. Furthermore, we show that genetic knockdown of hemocyte JNK signaling or Eiger expression mimics the effects of TFAM knockdown and leads to a non-autonomous suppression of body size without altering hemocyte numbers. Our data suggest that modulation of hemocyte mitochondrial metabolism can determine their non-autonomous effects on organismal growth by altering cytokine and systemic insulin signaling. Given that nutrient availability can control mitochondrial metabolism, our findings may explain how macrophages function as nutrient-responsive regulators of tissue and whole-body physiology and homeostasis.

## Teaser

**Summary:** This paper shows how changes in mitochondrial metabolism in larval blood cells can non-autonomously control overall body growth and development.

## INTRODUCTION

As animals develop, they need to coordinate growth across all their organs to ensure the proper attainment of functional body size. In most metazoans, this coordination relies on networks of organ-to-organ communication and endocrine signaling (Boulan et al., 2015; Droujinine and Perrimon, 2016; Texada et al., 2020). Defects in these networks and signaling pathways can impair development, leading to growth disorders and lethality.

The versatility of *Drosophila* genetics has led them to become a valuable model system for deciphering the tissue-to-tissue signaling networks that control body growth (Andersen et al., 2013; Grewal, 2012; Texada et al., 2020). In *Drosophila*, this growth occurs during the larval stage of development and is controlled by two main endocrine systems - insulin signaling and steroid ecdysone signaling - which determine both the rate of body growth and the timing of larval maturation (Danielsen et al., 2013; Koyama et al., 2020). *Drosophila* contains eight insulins (termed *Drosophila* insulin-like peptides, dILPs), of which three (dILPs 2,3 and 5) are expressed and secreted from a cluster of neurosecretory cells in the brain termed insulin-producing cells (IPCs). These dILPs can circulate through hemolymph and stimulate cell, tissue and body growth by binding to a cell surface insulin receptor and activating a conserved PI3K/Akt kinase signaling pathway (Grewal, 2009). Ecdysone is a steroid hormone produced and secreted from the prothoracic gland (PG). Short pulses of ecdysone secretion are essential for timing the larval molts through early larval development. In contrast, a final, larger ecdysone pulse triggers larval maturation to the pupal stage (Yamanaka et al., 2013). Several larval tissues can communicate with the IPC and the PG through secreted factors and cytokines to control the production and release of the dILPs and ecdysone (Texada et al., 2020). In many cases, these tissues function as sensors of environmental factors - such as nutrition, pathogens, toxins, and oxygen - and, in turn, signal to the brain and PG to couple insulin and ecdysone production to these external changes (Koyama et al., 2020). These mechanisms of inter-organ communication allow larvae to appropriately tailor their growth and development rate to fluctuations in their environmental conditions.

*Drosophila* hemocytes are macrophage like-cells that can control whole-body physiology and homeostasis (Banerjee et al., 2019). Like mammalian macrophages, they engulf damaged or dying cells or pathogens, most often in the context of innate immune responses (Gold and Bruckner, 2015; Parsons and Foley, 2016; Wang et al., 2014). However, recent studies have emphasized their importance as regulators of organismal physiology outside of immune responses. For example, genetic depletion of hemocytes in larvae impairs growth and development and can lead to lethality (Arefin et al., 2015; Charroux and Royet, 2009; Defaye et al., 2009; Preethi et al., 2020; Shia et al., 2009; Shin et al., 2020; Stephenson et al., 2022). These effects are due, in part, to reduced systemic insulin signaling and altered nutrient storage (Shin et al., 2020). In addition, hemocyte numbers are modulated by external factors such as nutrition, oxygen levels, infection, and odorants, which may provide one way that larvae couple changes in these environmental factors to control their development and homeostasis (Bakopoulos et al., 2020; Cho et al., 2018). For example, starvation-mediated decreases in hemocyte numbers are required for larvae to survive in poor nutrient conditions (Ramond et al., 2020). The ability of hemocytes to impact whole-body responses relies mainly on their ability to communicate with other tissues through cytokine and secreted signaling molecules. For example, hemocytes can express and secrete upd3, a cytokine similar to mammalian interleukin 6. In larvae, hemocyte-derived upd3 can act on the fat body to suppress insulin signaling (Shin et al., 2020) and on the PG to suppress ecdysone production and delay development (Romao et al., 2021). In adults, hemocyte-derived upd3 mediates the impairment of systemic glucose metabolism and impaired lifespan caused by a high-fat diet (Woodcock et al., 2015). Another hemocyte-secreted factor, pvf2, a fly homolog of the PDGF/VEGF growth factors, can act on the PG to suppress ecdysone production and delay larval maturation in low nutrient conditions (Juarez-Carreño and Geissmann, 2023 preprint). In addition, another hemocyte-expressed pvf ligand, pvf3, can signal to the fat body to control lipid storage (Cox et al., 2021a). These findings in *Drosophila* parallel those from mice where tissue-resident macrophages have been shown to influence both local and whole-body systemic metabolism (Cox and Geissmann, 2020; Cox et al., 2021b; Jaitin et al., 2019; Okabe and Medzhitov, 2016; Pirzgalska et al., 2017).

These studies in flies and mammals emphasize the critical role that macrophages play in maintaining tissue and whole-body homeostasis beyond their phagocytic roles in immune responses to infection and tissue damage. However, little is known about macrophage metabolic responses that are important for regulating these systemic effects. Recent studies on immunity and infection in mice have shown that mitochondrial metabolic reprogramming of macrophages can determine their cytokine expression and immune responses (Ryan and O'Neill, 2020). For example, activated macrophages use mitochondrial-derived metabolites such as succinate and citrate to control the expression of interleukins and cytokines to mediate their inflammatory and immune roles (Tannahill et al., 2013; Williams and O'Neill, 2018). In this paper, we have explored whether mitochondrial metabolism in *Drosophila* hemocytes impacts their numbers and effects on systemic physiology and growth.

## RESULTS

### Lowering OxPhos activity in hemocytes leads to reduced hemocyte numbers

**Fig. 1.**
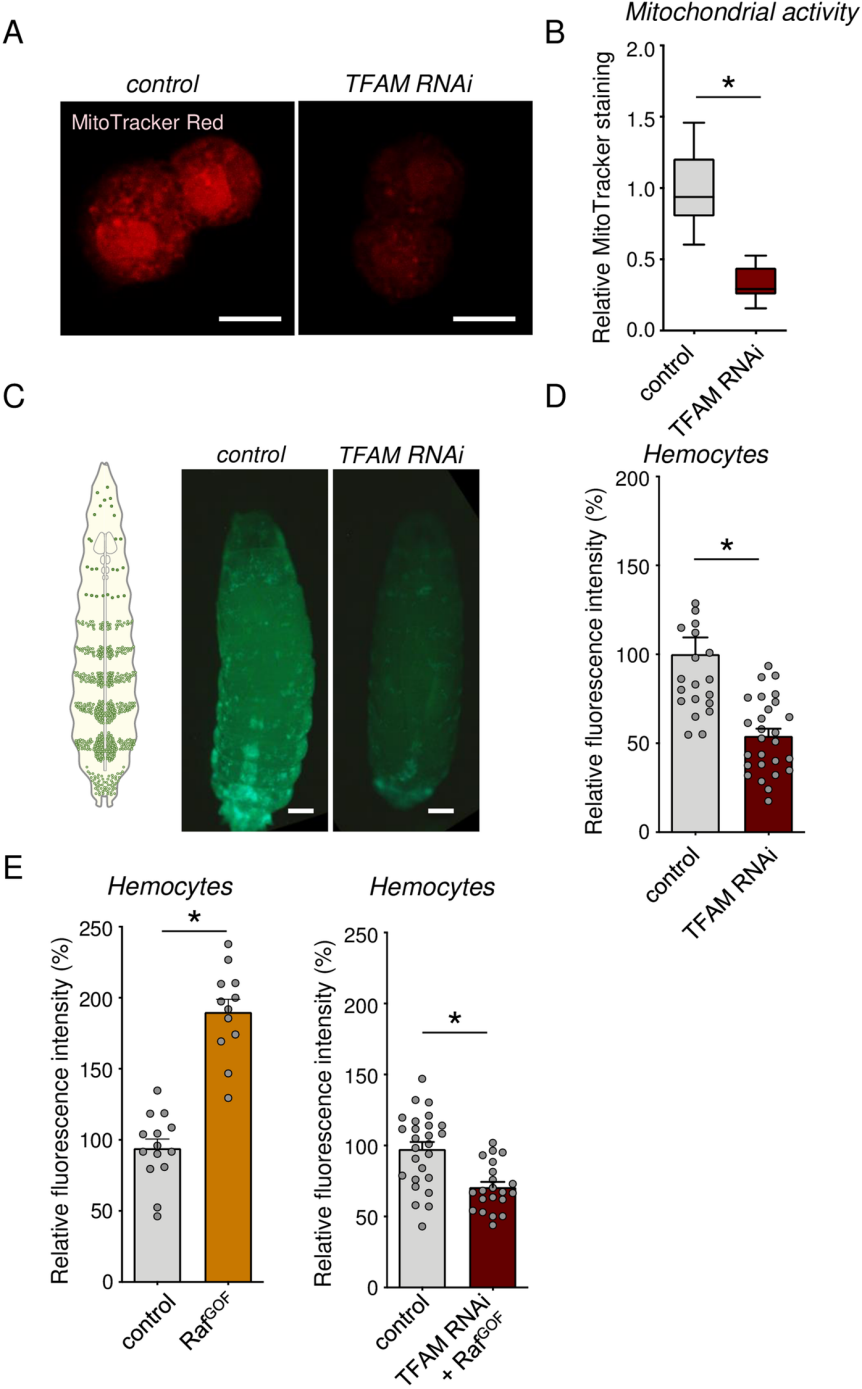
**Low bioenergetic activity in hemocytes leads to reduced hemocyte proliferation.** (A) Representative confocal micrographs of hemocyte mitochondria from control (*hml>+*) versus TFAM RNAi (*hml>UAS-TFAM-RNAi*) larvae at 96 h AEL stained with MitoTracker Red. Scale bars: 10 µm. (B) Quantification of MitoTracker Red staining intensity in A. Data are presented as box plots (25%, median and 75% values) with error bars indicating the minimum and maximum values (*P<0.05, unpaired *t*-test). Number (*n*) of samples: 21 (control) and 25 (TFAM-RNAi). (C) Representative images of hemocytes labeled with GFP from control (*hml>UAS-GFP*) versus TFAM RNAi (*hml>UAS-GFP, UAS-TFAM-RNAi*) larvae at wandering stage (∼144 h AEL). Scale bars: 100 µm. (D) Quantification of GFP fluorescent intensity in C. Data are represented as mean±s.e.m., with individual data points plotted as symbols (*P<0.05, unpaired *t*-test). *n* of samples: 21 (control) and 27 (TFAM-RNAi). See also Fig. S1A. (E) Quantification of relative fluorescent intensity of GFP-labelled hemocytes in control (*hml >UAS-GFP*) versus Raf^GOF^ (*hml>UAS-GFP, UAS-Raf^GOF^*) and control (*hml>UAS-GFP*) versus Raf^GOF^ combined with TFAM-RNAi (*hml>UAS-GFP, UAS TFAM-RNAi, UAS-Raf^GOF^*). Data are represented as mean±s.e.m., with individual data points plotted as symbols (*P<0.05 and ns, not significant, unpaired *t*-test). *n* of samples: 14 (control) versus 12 (Raf^GOF^) and 26 (control) versus 21 (TFAM-RNAi+Raf^GOF^).

To explore the effects of altered mitochondrial activity on hemocyte function, we used RNAi to knock down the mitochondrial transcription factor A, TFAM. TFAM is a nuclear-encoded transcription factor that localizes to mitochondria to transcribe the mitochondrial genome, including essential electron transport chain components. As a result, TFAM knockdown leads to reduced mitochondrial gene expression and OxPhos activity (Hamanaka et al., 2016; Sriskanthadevan-Pirahas et al., 2022). We used the hemocyte driver *hml-Gal4* to direct a *UAS-TFAM RNAi* transgene specifically in the hemocytes. This led to a strong reduction in hemocyte TFAM mRNA levels, without affecting whole-body TFAM levels, and also reduced hemocyte expression of two mitochondrial genome-encoded genes, mt:ATPase6 and mt:CoIII, that are both regulated by TFAM (Fig. S1A,B). We found that TFAM knockdown reduced the intensity of MitoTracker Red staining in isolated hemocytes, indicating reduced mitochondrial activity (Fig. 1A,B). We also saw that TFAM knockdown led to a significant reduction in hemocyte numbers (Fig. 1C,D), an effect also seen with a second independent UAS-TFAM RNAi line (Fig. S2A). In addition, TFAM knockdown prevented the increase in hemocyte numbers seen with the expression of an activated form of Raf kinase, a component of the oncogenic Ras signaling pathway (Fig. 1E). Importantly, we saw that TFAM knockdown did not affect the GFP intensity in individual hemocytes (Fig. S1C), indicating that the decrease in GFP-labelled hemocyte cell numbers was not simply due to a dampening of GFP expression in individual hemocytes. Our results demonstrate that lowering mitochondrial bioenergetic activity through TFAM knockdown in hemocytes can suppress hemocyte numbers even in the presence of activated Ras signaling.

### Hemocyte TFAM knockdown suppresses whole-body growth and development

**Fig. 2.**
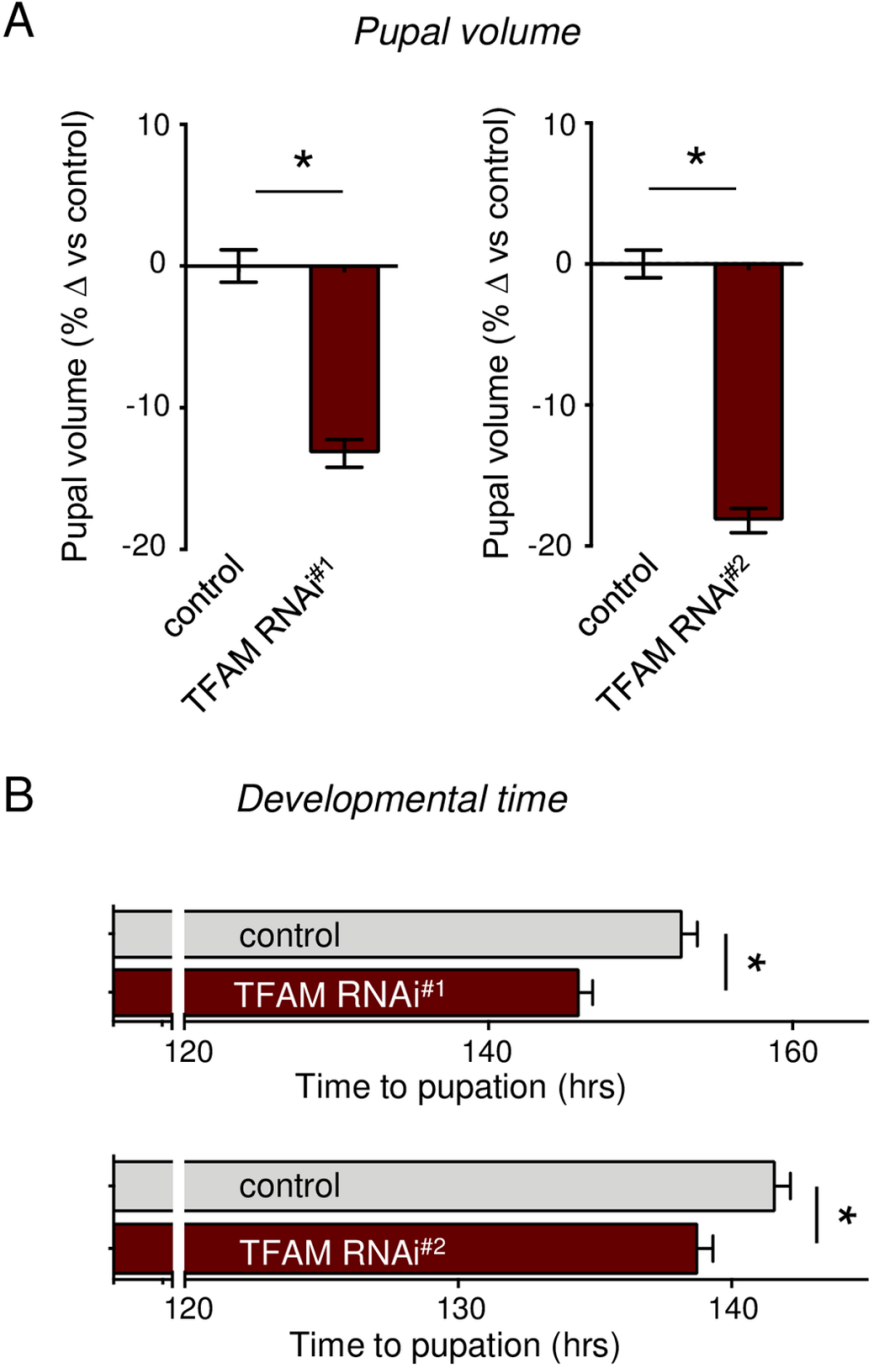
**Hemocyte TFAM knock down suppresses systemic growth and development.** (A) Relative change in pupal volume was calculated based on the average value of control (hml>+) animals. Data are presented as mean±s.e.m. (*P<0.05, Mann–Whitney *U-*test) for controls and two different TFAM RNAi lines (*hml>TFAM-RNAi*). Number (*n*) of pupae: 183 (control) versus 195 (TFAM RNAi^#1^) and 199 (control) versus 179 (TFAM RNAi^#2^). See also Fig. S1B. (B) Time to pupation was measured in control (*hml>+*) larvae versus larvae expressing one of two different TFAM RNAi transgenes (*hml>UAS- TFAM RNAi*). Data are presented as mean time to pupation±s.e.m. (*P<0.05, Mann–Whitney *U-*test). *n* of pupae: 171 (control) versus 135 (TFAM RNAi^#1^) and 179 (control) versus 107 (TFAM RNAi^#2^). See also Fig. S1C.

Previous studies have shown that changes in hemocyte function can impact whole-body physiology. We, therefore, examined whether alterations in mitochondrial bioenergetic activity might be important in these non-autonomous roles of hemocytes. We began by examining the effects on growth and development. We used RNAi to knock down TFAM in hemocytes and measured both time to pupation (as a measure of developmental rate) and pupal volume (as a measure of body size). We found that hemocyte TFAM knockdown using two independent RNAi lines led to a significant ∼15-20% reduction in pupal volume (Fig. 2A). When we measured developmental timing, we saw a significant but minimal decrease in time to pupation (only ∼3-6 h) in animals with hemocyte TFAM knockdown (Fig. 2B), which is unlikely to explain the substantial reduction in pupal size. No effect on pupal volume or developmental timing was seen with transgenic flies carrying UAS-TFAM RNAi transgene alone (Fig. S2B,C). We also saw that hemocyte TFAM knockdown did not alter larval feeding rate as assayed by the frequency of mouth hook movements (Fig. S2D). Hence, our results demonstrate that lowering mitochondrial bioenergetic activity in the larval hemocytes suppresses body size by reducing overall larval growth rate.

### Hemocyte TFAM knockdown suppresses systemic insulin signaling

**Fig. 3.**
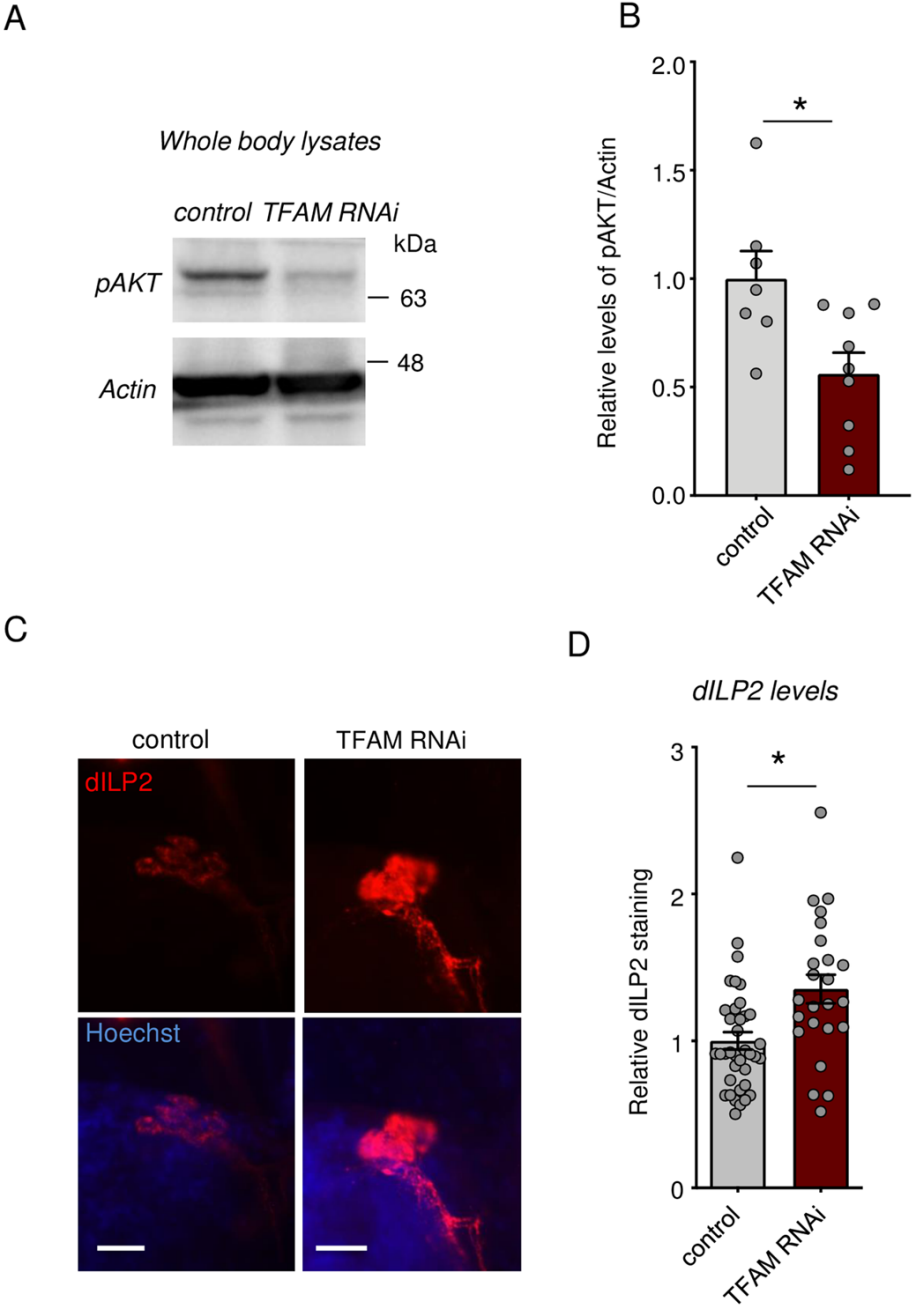
**Hemocyte TFAM knock suppresses systemic insulin signaling by inhibiting dILP2 secretion from brain IPCs.** (A) Western blots of whole-body samples from control (*hml>+*) versus TFAM RNAi (*hml>UAS-TFAM-RNAi*) larvae at 96 h AEL analyzed using Phospho-Akt and actin antibodies. (B) Quantification of Western blots from A. Data are relative levels of phospho-Akt band intensity corrected for actin band intensity. Data are presented as box plots (25%, median and 75% values) with error bars indicating the minimum and maximum values [*P<0.05 unpaired *t*-test, *n*=7 (control) and 9 (TFAM RNAi) groups per condition with 20 larvae in each group]. (C) Representative images for brain IPCs stained with dILP2 in control (*hml>+*) versus TFAM RNAi (*hml>UAS-TFAM-RNAi*) larvae at 96 h AEL larvae. Scale bars: 20 µm. (D) Quantification of relative dILP2 fluorescent intensity in (C). Data are represented as mean±s.e.m., with individual data points plotted as symbols (*P<0.05, unpaired *t*-test). *n* of samples: 37 (control) and 24 (TFAM-RNAi).

We next explored how hemocyte TFAM knockdown might suppress overall body growth. One of the central regulators of growth in larvae is the endocrine insulin pathway. Flies have eight *Drosophila* insulin-like peptides (dILPs), seven of which (dILP1-7) have been shown to play growth-promoting roles (Brogiolo et al., 2001; Ikeya et al., 2002), while dILP8 primarily plays a role in controlling tissue-damage-induced delays in larval development (Gontijo and Garelli, 2018). dILPs 1-7 bind to a single insulin receptor and activate a conserved PI3 Kinase/Akt kinase signaling pathway that can stimulate growth in all larval tissues. We found that hemocyte knockdown of TFAM led to reduced levels of whole-body phosphorylated Akt (Fig. 3A,B), consistent with reduced systemic insulin signaling. One primary way insulin signaling is controlled is through the production and release of three dILPs (2, 3 and 5) from the insulin-producing cells (IPCs) in the brain (Grewal, 2009). We found that hemocyte TFAM knockdown did not affect the whole-body mRNA levels of any of the seven growth-promoting dILPs (Fig. S3). However, when we used dILP2 antibody staining to examine the IPCs, we saw an accumulation of dILP2 protein (Fig. 3C,D), an effect characteristic of dILP2 retention due to reduced dILP2 secretion (Geminard et al., 2009). Taken together, our results suggest that one way that hemocyte-specific knockdown of TFAM leads to reduced body growth is by lowering brain-derived dILP2 secretion leading to suppressed systemic insulin signaling.

### TFAM knockdown inhibits hemocyte JNK signaling, and genetic suppression of hemocyte JNK signaling suppresses body growth

**Fig. 4.**
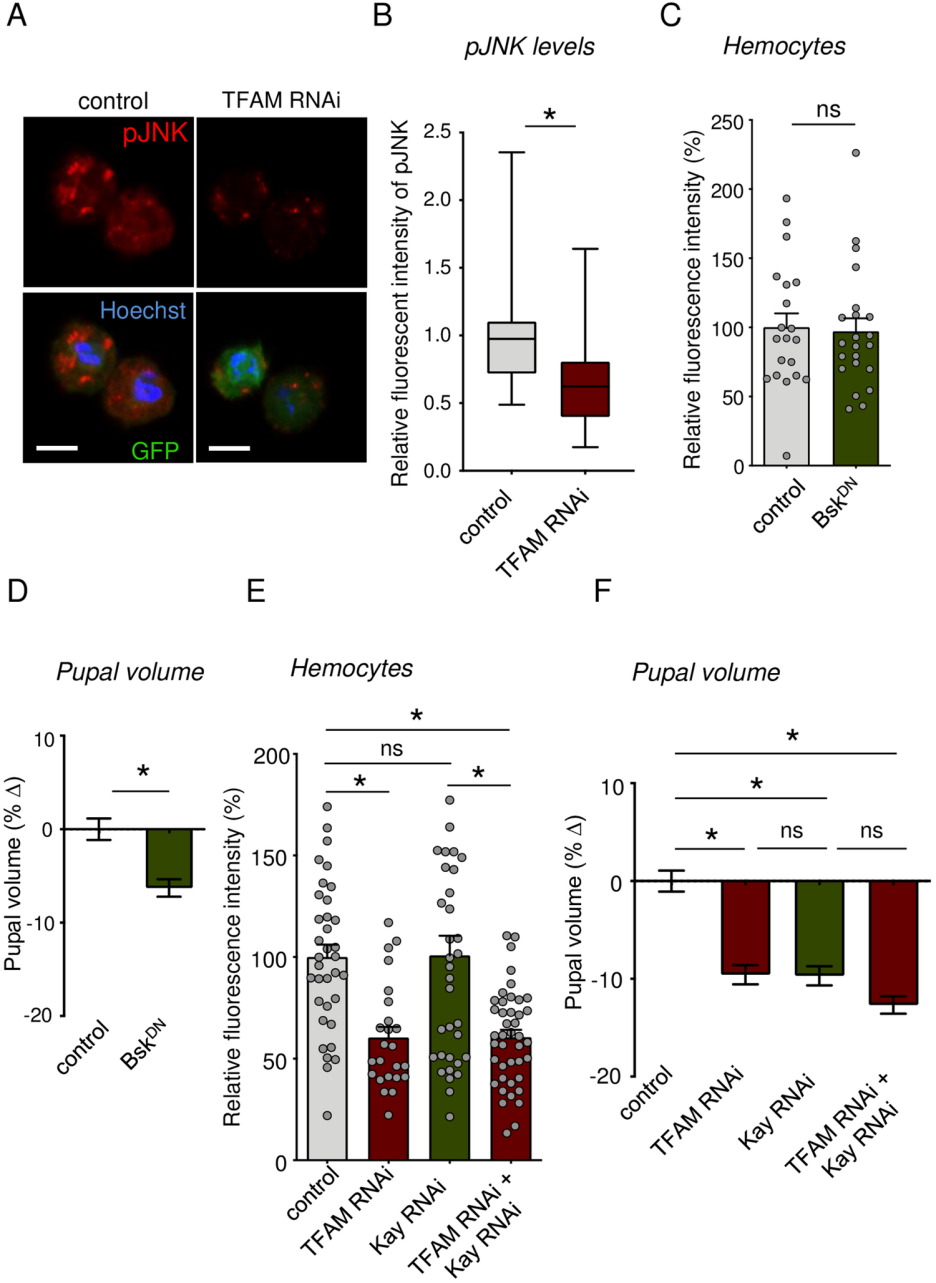
**Hemocyte specific knock down of JNK signaling suppresses systemic growth.** (A) Representative images for hemocytes stained with phospho-JNK (pJNK) in control (*hml>+*) versus TFAM RNAi (*hml>UAS-TFAM-RNAi*) larvae at 96 h AEL larvae. The scale bars represent 5 µm. (B) Quantification of relative pJNK fluorescent intensity in hemocytes (A). Data are presented as box plots (25%, median and 75% values) with error bars indicating the minimum and maximum values (*P<0.05, unpaired *t*-test). *n* (# of hemocytes)=36 (control) and 36 (TFAM-RNAi). (C) Quantification of relative fluorescent intensity of GFP-labelled hemocytes in control (*hml>+*) versus Bsk^DN^ (*hml>Bsk^DN^*). Data are represented as mean±s.e.m., with individual data points plotted as symbols (*P<0.05 and ns, not significant, unpaired *t*-test). Number (*n*) of samples: 20 (control) and 22 (Bsk^DN^). (D) Relative change in pupal volume was calculated based on the average value of control (*hml>+*) animals. Data are presented as mean±s.e.m. (*P<0.05 and ns, not significant, Mann–Whitney *U-*test) for control (*hml>+*) versus Bsk^DN^ (*hml>Bsk^DN^*) animals. *n* of pupae: 206 (control), 206 (Bsk^DN^). (E) Quantification of relative fluorescent intensity of GFP-labelled hemocytes in control (*hml>+*), TFAM RNAi (*hml>UAS-TFAM RNAi*), Kay RNAi (*hml>UAS-Kay RNAi*) and TFMA RNAi+Kay RNAi (*hml>UAS-TFAM RNAi+UAS-Kay RNAi*) larvae. Data are represented as mean±s.e.m., with individual data points plotted as symbols (*P<0.05 and ns, not significant, unpaired *t*-test). *n* of samples: 35 (control), 35 (*TFAM RNAi*), 24 (*Kay RNAi*), and 42 (*TFAM RNAi+Kay RNAi*). (F) Relative change in pupal volume was calculated based on the average value of control (hml>+) animals. Pupal volume data analysis in control (*hml>+*), TFAM RNAi (*hml>UAS-TFAM RNAi*), Kay RNAi (*hml>UAS-Kay RNAi*) and TFMA RNAi+Kay RNAi (*hml>UAS-TFAM RNAi+UAS-Kay RNAi*) larvae. Data are represented as mean±s.e.m., with individual data points plotted as symbols (*P<0.05 and ns, not significant, unpaired *t*-test). *n* of samples: 180 (control), 202 (*TFAM RNAi*), 213 (*Kay RNAi*), and 200 (*TFAM RNAi+Kay RNAi*).

We next wanted to investigate the downstream effects of TFAM knockdown in hemocytes. Alterations in mitochondrial function have been shown to modulate JNK signaling pathway activity in *Drosophila* (Borch Jensen et al., 2017). We therefore examined phosphorylation levels of JNK. We found hemocytes with TFAM knockdown have significantly lower pJNK staining (Fig. 4A,B), suggesting that lowering mitochondrial OxPhos suppresses the JNK signaling pathway. To explore whether this decrease in JNK signaling might explain the effects of hemocyte TFAM knockdown on both hemocyte numbers and body growth, we genetically inhibited JNK pathway activity in hemocytes by expression of either a dominant negative version of the JNK kinase, *Basket (BskDN)*, or by RNAi-mediated knockdown of *Kayak (Kay RNAi)*, a transcriptional target and effector of JNK signaling in flies. In both cases, we saw that inhibition of JNK signaling in hemocytes had no effects on hemocyte numbers (Fig. 4C,E), suggesting that suppression of JNK signaling does not explain the reduced hemocyte numbers following TFAM knockdown. However, we saw that hemocyte-specific expression of either *BskDN* or hemocyte *Kay RNAi* led to a reduction in pupal volume (Fig. 4D,F) and that *Kay RNAi* had little effect on time to pupation (Fig. S4A). In addition, we saw that the reduction in body size following hemocyte-specific knockdown of TFAM and Kay was comparable to the effects of either knockdown alone, suggesting both factors function similarly (Fig. 4F). Together, our results suggest that one way that the reduction of OxPhos activity by TFAM knockdown suppresses whole-body growth via reduced activity of the JNK-signaling pathway and that these effects are independent of any changes in hemocyte number.

### Mitochondrial OxPhos controls hemocyte Eiger expression and can regulate body size

**Fig. 5.**
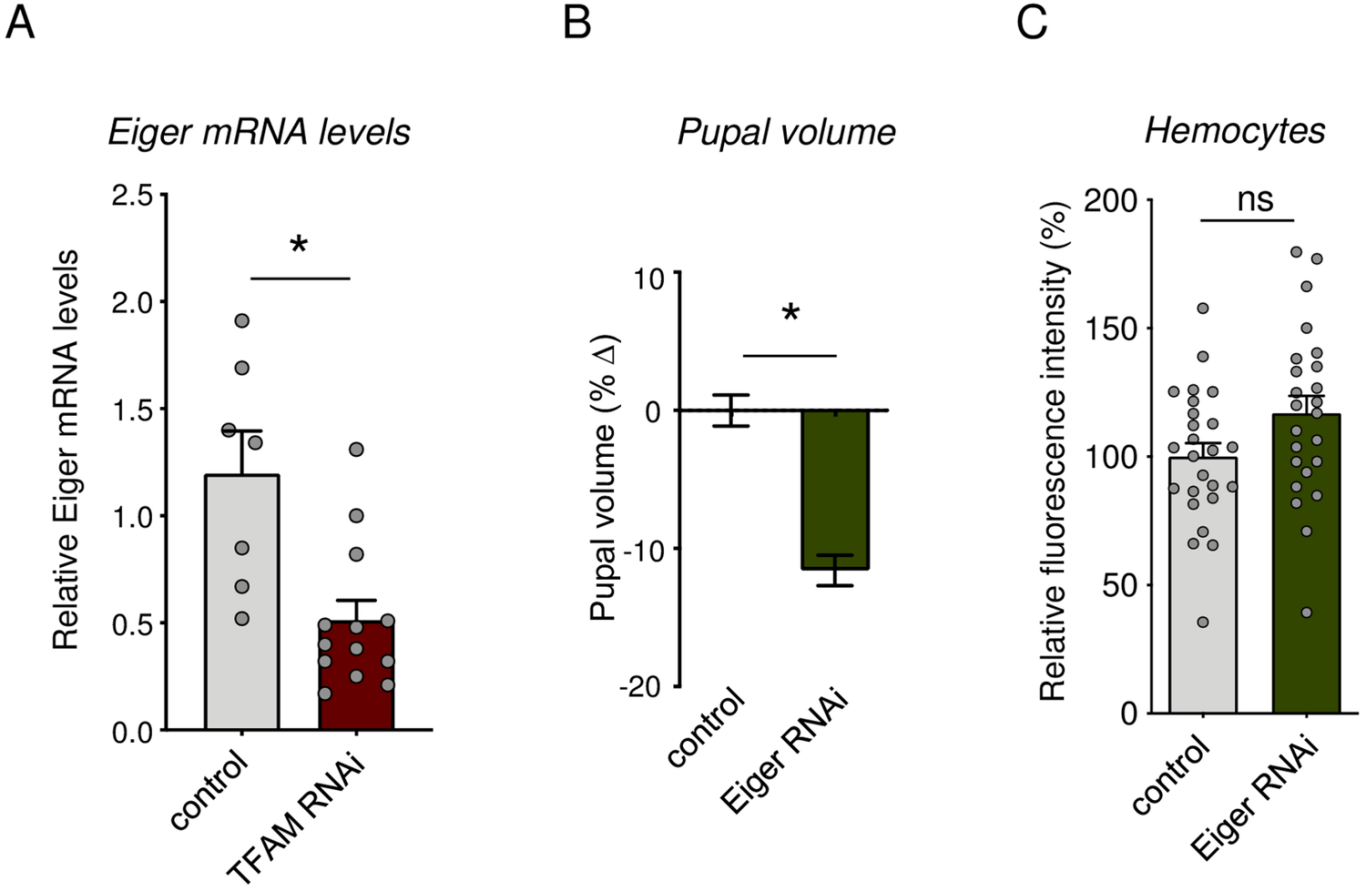
**Hemocyte specific cytokine knock down suppresses body growth.** (A) Hemocyte specific Eiger mRNA levels measured by qRT-PCR in control (*hml>+*) versus TFAM RNAi (*hml>UAS-TFAM-RNAi*) larvae at 120 h AEL. Data are represented as mean±s.e.m., with individual data points plotted as symbols (*P<0.05 and ns, not significant, unpaired *t*-test). Number (*n*) of samples: 7 (control) and 13 (TFAM RNAi). (B) Relative change in pupal volume of control (*hml>+*) versus Eiger RNAi (*hml>UAS-Eiger RNAi*) larvae. Data are presented as mean±s.e.m. (*P<0.05 and ns, not significant, Mann–Whitney *U-*test). *n* of pupae: 187 (control), 165 (Eiger RNAi). (C) Quantification of relative fluorescent intensity of GFP-labelled hemocytes in control (*hml>+*) versus Eiger RNAi (*hml>UAS-Eiger RNAi*) larvae. Data are represented as mean±s.e.m., with individual data points plotted as symbols (*P<0.05 and ns, not significant, unpaired *t*-test). *n* of samples: 25 (control) and 24 (Eiger RNAi).

Hemocytes express many different secreted factors that can impact whole-body physiology. Interestingly, we observed a significant reduction in hemocyte mRNA levels of one such factor – the TNF alpha homolog, Eiger – following TFAM knockdown in hemocytes (Fig. 5A). We also saw that RNAi- mediated Eiger knockdown in hemocytes led to smaller body size (Fig. 5B) without any effect on hemocyte number (Fig. 5C). Flies carrying just the UAS-Eiger RNAi transgene alone showed no significant change in body size (Fig. S4B) These results suggest that one way that lowered hemocyte mitochondrial OxPhos activity suppresses body growth is through altered cytokine signaling.

## DISCUSSION

**Fig. 6.**
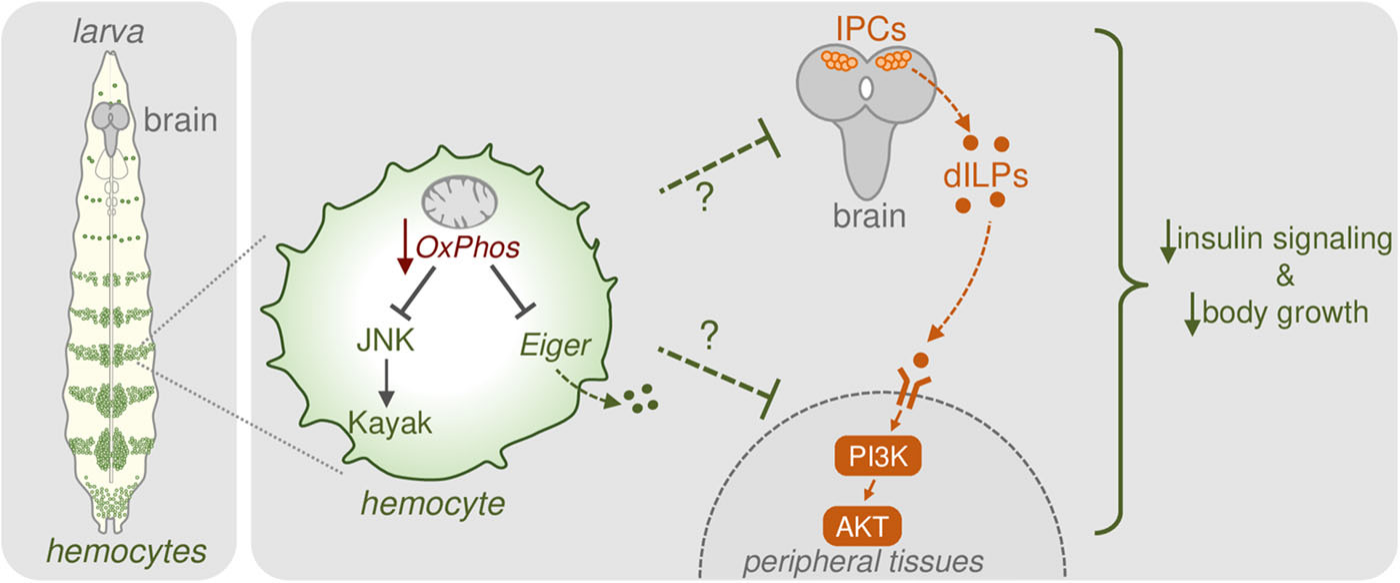
**Low bioenergetic mitochondrial activity in hemocyte leads to suppression of systemic insulin signaling.** When hemocyte mitochondrial OxPhos activity is low (for example following TFAM knockdown), expression of Eiger and the activity of the JNK pathway are reduced. Under these conditions, dILP2 secretion from the brain IPC cells and systemic insulin signaling are reduced leading to reduced animal growth and development.

Our main finding is that suppressing hemocyte mitochondrial OxPhos activity can exert both autonomous and non-autonomous effects on growth (Fig. 6). The autonomous effects involve suppressing hemocyte numbers even when proliferation is stimulated by activation of the oncogenic Ras pathway. This result is consistent with similar studies in mouse models of lung cancer showing that mitochondrial metabolism is essential for Ras-mediated tumors (Hamanaka et al., 2016). The non-autonomous effect of reduction in hemocyte OxPhos was suppression in overall body growth. The final pupal volume was reduced, but developmental timing was only modestly accelerated. Therefore, we likely saw decreased body size resulting from reduced overall growth rather than an acceleration of the growth period. Consistent with this, we saw that the decrease in body growth caused by hemocyte TFAM knockdown was accompanied by reduced whole-body Akt phosphorylation and reduced dILP2 release from the IPCs in the brain, pointing to a reduction in systemic insulin signaling, the primary regulator of body growth. The mechanisms by which reduced hemocyte TFAM causes a non-autonomous decrease in systemic insulin signaling remain unclear. The brain can integrate signals from several different tissues to control the expression and release of dILPs from the IPCs. Furthermore, hemocytes express an array of secreted factors that can act on remote tissues to control their physiology. Hence, changes in hemocyte-derived secreted factors may mediate the non-autonomous effects we see with hemocyte TFAM.

One potential effector of reduced OxPhos activity in hemocytes is the reduction of JNK signaling. We saw that TFAM knockdown leads to reduced phosphorylation of JNK and that genetic suppression of JNK signaling in hemocytes could mimic the effects of TFAM knockdown on reduced body size. Interestingly, the effects of JNK pathway suppression and TFAM knockdown on body size were not additive, suggesting they function in the same pathway. Our work also identified the hemocyte-expressed secreted factor, Eiger, as a potential link between changes in hemocyte OxPhos activity and the control of body growth. We saw that TFAM knockdown decreased Eiger mRNA expression in hemocytes and that hemocyte knockdown of Eiger mimicked the effects of TFAM knockdown and led to a reduction in body size. These results suggest that reduced hemocyte OxPhos activity may decrease Eiger expression to mediate non-autonomous effects on body size. Interestingly, fat-derived Eiger can inhibit dILP secretion from IPCs (Agrawal et al., 2016). Hence, it may appear paradoxical that blocking hemocyte production of Eiger would lead to reduced body size. However, it is possible that the effects on body size we see with Eiger manipulation either occur independently of changes in systemic insulin signaling, or that the impact of Eiger on systemic insulin signaling may depend on the cell type it is expressed from (fat versus hemocytes). Further experiments exploring how hemocyte-derived Eiger and JNK signaling affect systemic insulin signaling will help clarify these possibilities.

An interesting finding from our work was that the body size suppression caused by hemocyte suppression of JNK signaling or Eiger knockdown was independent of any change in hemocyte number. This suggests that the non-autonomous effects of hemocyte OxPhos reduction on body growth may not be because of changes in hemocyte number. Similarly, a recent report showed that the impact of hemocytes on developmental timing mediated by pvf2 signaling in response to low nutrients was also independent of hemocyte cell number (Juarez-Carreño and Geissmann, 2023 preprint). In contrast, another study reports that a reduction in hemocyte numbers is needed for their effects on nutrient storage and survival in poor nutrients (Ramond et al., 2020). Thus, both the metabolic status and number of hemocytes are important for determining their impact on organismal physiology.

A question prompted by our work is how does a reduction in hemocyte mitochondrial OxPhos activity lead to suppression of JNK signaling and Eiger expression? Given that Eiger is known to signal via upregulation of JNK signaling (Agrawal et al., 2016; Andersen et al., 2015; Kanda et al., 2011; Sanchez et al., 2019), it is possible that the reduction in hemocyte JNK signaling that we see following TFAM knockdown may be occurring because of decreased autocrine Eiger function. Another possibility is that these effects are caused by alterations in reactive oxygen species (ROS) levels. The mitochondrial electron transport chain is a major source of ROS production in cells, and JNK is stimulated by ROS levels (Gera et al., 2022; Santabarbara-Ruiz et al., 2015). Thus, TFAM knockdown, by lowering OxPhos activity, may limit ROS production and thereby reduce JNK activity. Additionally, a reduction in OxPhos may reprogram mitochondrial metabolism leading to alterations in TCA cycle intermediates. Changes in the levels of these metabolites, such as succinate and citrate, have previously been shown to couple mitochondrial metabolism in activated mammalian macrophages to the expression of cytokines by altering the activity of chromatin modifiers (Ryan and O'Neill, 2020; Van den Bossche et al., 2017). Hence, a similar mechanism may regulate Eiger expression in hemocytes. It is also possible that TFAM knockdown modulates hemocyte glycolytic metabolism. We previously showed that lowering TFAM levels in the larval fat body increased glycolytic gene expression. Moreover, in *Drosophila,* upregulation of hemocyte glycolysis has been shown to promote changes in systemic metabolism (Krejcova et al., 2019) upon infection and in mice, upregulation of macrophage glycolysis is essential for their activation and cytokine secretory functions (Viola et al., 2019). To explore these possibilities, future experiments can examine whether manipulation of mitochondrial or glycolytic metabolism can mimic TFAM knockdown and lead to reduced JNK activity and Eiger expression and whether restoration of JNK activity and/or Eiger expression can either wholly or partially reverse the effects of TFAM knockdown on insulin signaling, body growth and development.

Macrophages play multiple essential roles in regulating tissue and whole-body metabolic homeostasis (Cox and Geissmann, 2020; Cox et al., 2021b; Jaitin et al., 2019; Okabe and Medzhitov, 2016; Pirzgalska et al., 2017). These general regulatory roles of macrophages are seen in both invertebrates and vertebrates and are often influenced by changes in nutrient availability (Cox et al., 2021a,b). Given that regulation of mitochondrial metabolism is a downstream target of many conserved nutrient-responsive signaling pathways (de la Cruz Lopez et al., 2019), our findings suggest that changes in mitochondrial metabolism may link the nutrient-sensing properties of macrophages to their role as regulators of metabolic homeostasis.

## MATERIALS AND METHODS

### *Drosophila* food and genetics

Flies were raised on a medium containing 150 g agar, 1600 g cornmeal, 770 g Torula yeast, 675 g sucrose, 2340 g D-glucose, and 240 ml acid mixture (propionic acid/phosphoric acid) per 34 L water and maintained at 25°C. For all GAL4/UAS experiments, homozygous GAL4 lines were crossed to the relevant UAS line(s), and the larval progeny were analyzed. Control animals were obtained by crossing the appropriate homozygous GAL4 line to flies of the same genetic background as the relevant experimental UAS transgene line. All experiments were performed with mixed groups of male and female larvae.

### *Drosophila* strains

The following strains were used: *w^1118^*, *hml-GAL4, UAS-GFP, UAS-Eiger RNAi* (VDRC 108814), GD control line (60000 TK), KK control line (VDRC 60100 TK), UAS-Kayak-RNAi (VDRC 6212 GD), *UAS-TFAM RNAi #1 (4217R-2), UAS-TFAM RNAi #2* (4217R-3 -Fly Stocks of National Institute of Genetics - NIG-FLY).

### Measurement of *Drosophila* developmental time

For measuring development timing to the pupal stage, newly hatched larvae were collected at 24 h AEL and placed in food vials (50 larvae per vial). The number of newly formed pupae was counted twice daily until all larvae had pupated.

### Pupal imaging and pupal volume measurement

Pupae were imaged using a Zeiss Discovery V8 Stereomicroscope with Axiovision imaging software. Pupal length and width were measured, and pupal volume was calculated using the formula: volume=4/3π(L/2) (l/2)2.

### Quantification of hemocyte number

To assay for hemocyte numbers, we used an *hml-GAL4, UAS-GFP*, to GFP-label hemocytes. Larvae were collected during the L3 wandering larval stage using forceps and cleaned by being placed in a small petri dish containing 5 mL of phosphate-buffered saline (PBS) for 30 s. The clean larvae were then transferred to another small petri dish and fluorescence-imaged using a ZEISS SteREO Discovery V8 microscope and ZEN imaging software (blue edition) at 8.0x magnification. Next, the NIH ImageJ software was used to quantify the fluorescence intensity in a defined region in posterior segments of each larvae where hemocytes are clustered. This value was then corrected for background autofluorescence by subtracting the average fluorescence intensity measured from unlabeled *w^1118^* L3 wandering larvae.

### MitoTracker Deep Red staining

Hemocytes from 96 h AEL larvae were collected and stained with MitoTracker Deep Red FM (1:1000 dilution of 1 mM, Molecular probes M22426) for 40 min and fixed at room temperature using 8% PFA for 30 min. After washing three times, cells were mounted on slides using Vecta Shield mounting medium. The mitochondrial images were acquired through a Zeiss confocal microscope LSM 880. Cytoplasmic MitoTracker Red staining intensity was quantified using Image J by measuring fluorescent intensity within a fixed area of the cytoplasm in cells of each experimental condition.

### Measurement of larval mouth hook contractions

The feeding behavior (mouth hook contractions) assay was conducted as outlined previously (Bhatt and Neckameyer, 2013). Briefly, age-matched third instar larvae were placed in the center of a tissue culture plate filled with agar and overlaid with 5 ml of 2% activated baker's yeast solution. Larvae were first allowed to acclimate for 30 s. Then, the number of mouth hook contractions was recorded for 60 s. Each plotted point represents the result from a single larva.

### Preparation of larval protein extracts

*Drosophila* larvae (96 h. AEL) were lysed with homogenization and sonication in a buffer containing 20 mM Tris-HCl (pH 8.0), 137 mM NaCl, 1mM EDTA, 25% glycerol, 1% NP-40 and with the following inhibitors: 50 mM NaF, 1 mM PMSF, 1mM DTT, 5 mM sodium ortho vanadate (Na_3_VO_4_) and Protease Inhibitor cocktail (Roche #04693124001) and Phosphatase inhibitor (Roche #04906845001), according to the manufacturer instructions.

### Western blotting, immunostaining and antibodies

Protein concentrations were measured using the Bio-Rad Dc Protein Assay kit II (Bio-Rad 5000112). Protein lysates (100 μg) were resolved by SDS-PAGE and electro transferred to a nitrocellulose membrane, subjected to Western blotting analysis with specific antibodies, and visualized by chemiluminescence (enhanced ECL solution Perkin Elmer). The primary antibodies used in this study were: anti-phospho-AKT-Ser505 (1:1000, Cell Signaling Technology #4054), anti-actin (1:1000, Santa Cruz Biotechnology, #sc-8432), anti-dILP2 (Texada et al., 2019) (1:500), and anti-phospho-JNK (1:500, Cell Signaling Technology #4668S). Goat and donkey secondary antibodies were purchased from Santa Cruz Biotechnology (#sc-2030, 2005, 2020).

### Larval brain staining

Larval brains were dissected and fixed for 30 min in 4% formaldehyde in PBS, washed three times in PBS with 0.1% Triton X-100 (PBT). Tissues were then pre-blocked in PBT+5% BSA+2% fetal bovine serum for 2 h and then incubated overnight at 4°C with the primary antibody (1:1000 dilution of anti-dILP2) in 5% BSA+ PBT, and then washed three times in PBT+0.5% BSA. A cocktail of secondary antibodies was then added to the block (final concentration 1:400) and the tissues were incubated overnight in secondary at 4°C. The samples were then washed three times for 15 min each time, with PBT+0.5% BSA and mounted on slides. Brains were imaged using a Zeiss Stereo Discovery V8 microscope using Axiovision software.

### Hemocyte RNA isolation

Hemocytes were collected from 20 larvae per sample at 120 h AEL. Five larvae were washed in PBS and placed onto paraffin film within a 5µl PBS droplet. While observing the larvae under the stereomicroscope, the posterior and anterior cuticles were disrupted using fine-pointed forceps and the hemolymph was allowed to flow onto the paraffin film. The pooled hemolymph from 20 larvae were collected in a microcentrifuge, and hemocytes were pelleted by centrifuging at 1000 ***g*** for 20 min at 4°C and snap frozen on dry ice. Total RNA was isolated from each sample using TRIzol according to the manufacturer's instructions.

### Quantitative RT-PCR measurements

Total RNA was extracted from hemocytes collected from 20 larvae at 120 h AEL or whole larval lysates at 96 h AEL using TRIzol according to the manufacturer's instructions (Invitrogen #15596-018). RNA samples isolated from the same number of larvae (control versus experimental) were DNase treated (Ambion #2238G) and reverse transcribed using Superscript II (Invitrogen #100004925). The generated cDNA was used as a template to perform qRT–PCRs (ABI 7500 real-time PCR system using SyBr Green PCR mix) using gene-specific primers. PCR data were normalized to RpL32. All primer sequences are listed in the Table S1.

### Statistical analysis

All qRT-PCR data and quantification of immunostaining data were analyzed by Student’s *t*-test, two-way ANOVA followed by *post-hoc* student's *t*-test, or Mann–Whitney *U-*test where appropriate. All statistical analysis and data plots were performed using GraphPad Prism statistical software (GraphPad Prism version 9.2.0 for Mac OS, GraphPad Software, Boston, Massachusetts USA, www.graphpad.com). Differences were considered significant when *P*-values were less than 0.05.

## Supplementary Material

10.1242/biolopen.059968_sup1 Supplementary information
