# Supplementary material for: Mitochondrial metabolism in Drosophila macrophage-like cells regulates body growth via modulation of cytokine and insulin signaling
Source: Biol Open. 2023 Nov 29;12(11):bio059968. doi: 10.1242/bio.059968 (PMC10695174; doi:10.1242/bio.059968)
Supplement: Supplementary information [file biolopen-12-059968-s1.pdf]

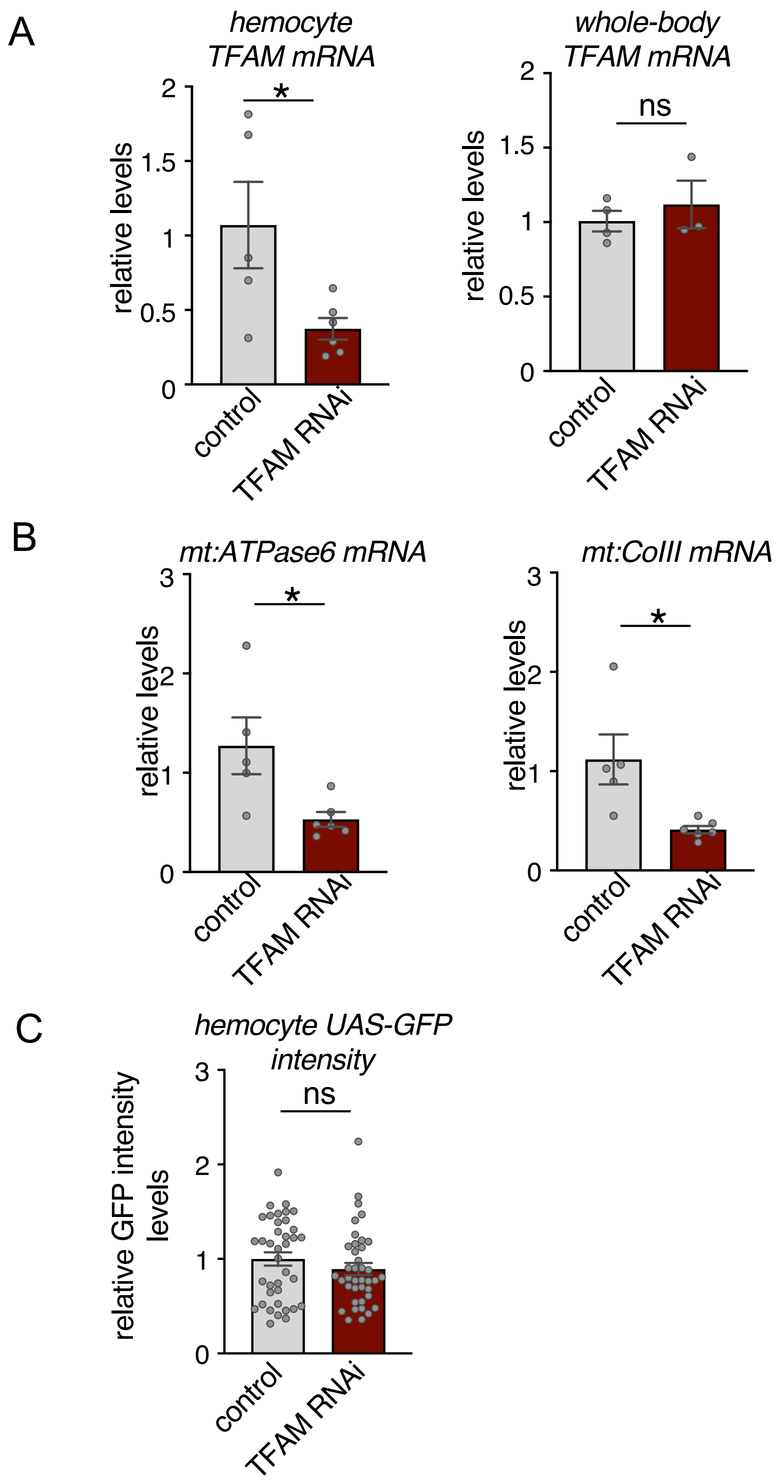

Fig. S1. TFAM knockdown in hemocytes suppresses hemocyte proliferation and systemic growth (related to Fig. 1 and 2)

(A) mRNA levels of TFAM measured by qRT-PCR in isolated hemocytes (left) or whole- body (right) samples from control (*hml* > +) versus TFAM RNAi (*hml* > *UAS-TFAM-RNAi*) larvae. Data are represented as mean ± SEM, with individual data points plotted as symbols (*p < 0.05 and ns, not significant, unpaired t-test).

(B) mRNA levels of two TFAM mitochondrial genome target genes, mt:ATPase6 and mt:CoIII measured by qRT-PCR in isolated hemocytes from control (*hml* > +) versus TFAM RNAi (*hml* > *UAS-TFAM-RNAi*) larvae. Data are represented as mean ± SEM, with individual data points plotted as symbols (*p < 0.05, unpaired t-test).

(**C**) Quantification of GFP fluorescent intensity of isolated hemocytes from control (*hml>UAS-GFP*) versus TFAM RNAi[#1] (*hml>UAS-GFP, UAS-TFAM-RNAi*) (*p < 0.05, unpaired t-test). Data are represented as mean ± SEM, with individual data points (relative GFP intensities of individual cells) plotted as symbols (ns, not significant, unpaired t-test). n (# of individual cells) = 38 per experimental group.

Biology Open • Supplementary information

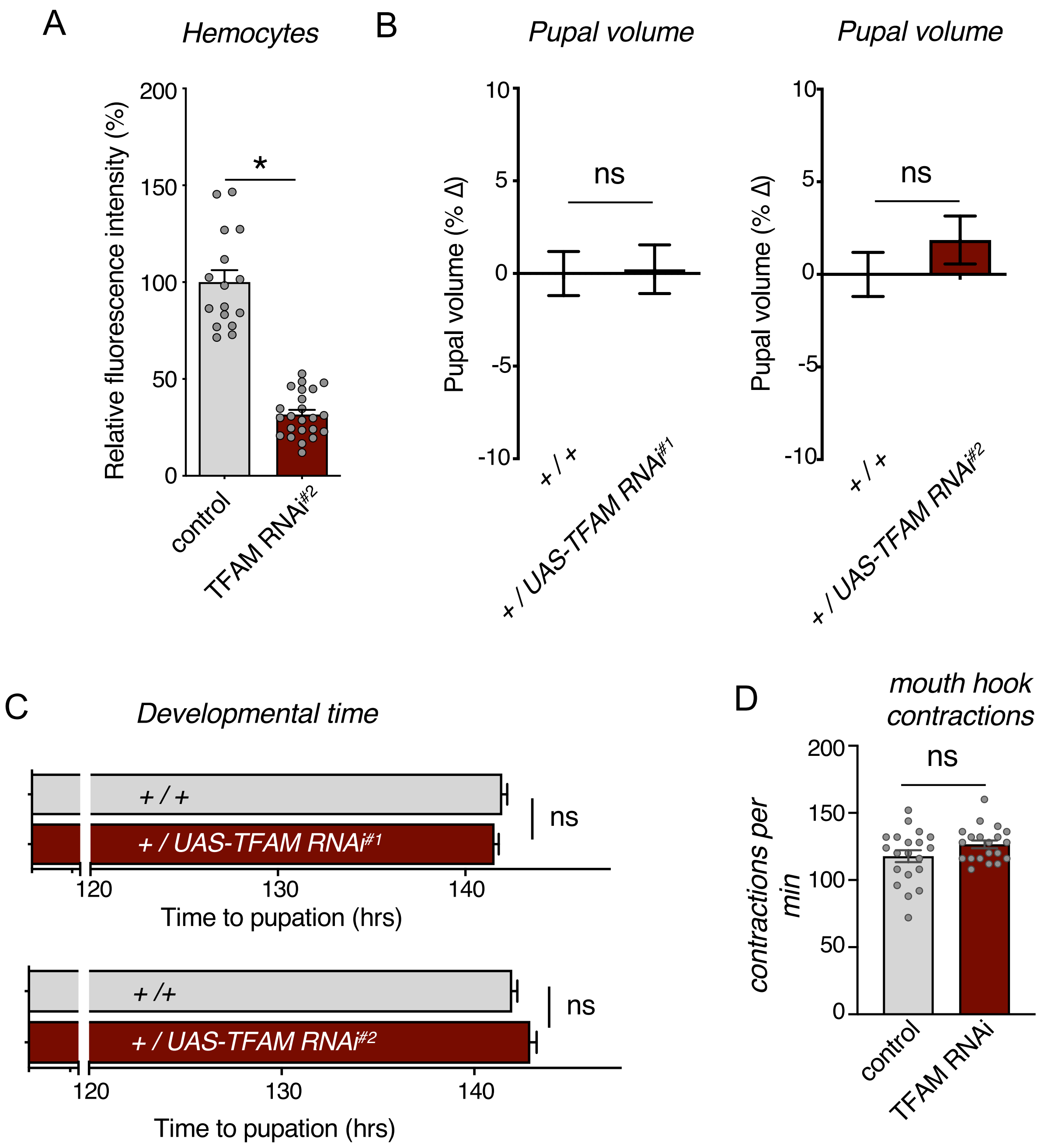

Fig. S2. TFAM knockdown in hemocytes suppresses hemocyte proliferation and systemic growth (related to Fig. 1 and 2)
(**A**) Quantification of GFP fluorescent intensity from control (*hml>UAS-GFP*) versus TFAM RNAi#1 (*hml>UAS-GFP, UAS-TFAM-RNAi*) larvae at wandering stage. Data are represented as mean ± SEM, with individual data points plotted as symbols (*p < 0.05, unpaired t-test). n (# of samples) = 16 (control) and 23 (TFAM-RNAi#1).
(**B**) Relative change in pupal volume was calculated based on the average value of control (+/+) animals. Data are presented as mean +/- SEM (*p < 0.05, Mann-Whitney U test) for controls and two different TFAM RNAi lines *(+/UAS-TFAM-RNAi*). n (# of pupae) = 147 (control) vs 148 (TFAM RNAi#1) and 147 (control) vs 146 (TFAM RNAi#2).
(**C**) Time to pupation was measured in control (+/+*)* larvae versus larvae expressing one of two different TFAM RNAi transgenes *(+/UAS- TFAM RNAi*). Data are presented as mean time to pupation +/- SEM (*p < 0.05, Mann-Whitney U test). n (# of pupae) = 590 (control) vs 675 (TFAM RNAi#1) and 590 (control) vs 592 (TFAM RNAi#2). **(D)** Mouth hook contractions in (*hml*>+) versus TFAM RNAi (*hml>UAS-TFAM-RNAi*) larvae at L3 stage. Data are represented as mean ± SEM, with individual data points plotted as symbols and reflecting mouth hook contractions per minute for individual larvae (ns = not significant, unpaired t-test).). n (# of larvae) = 20 per experimental group.

Biology Open • Supplementary information

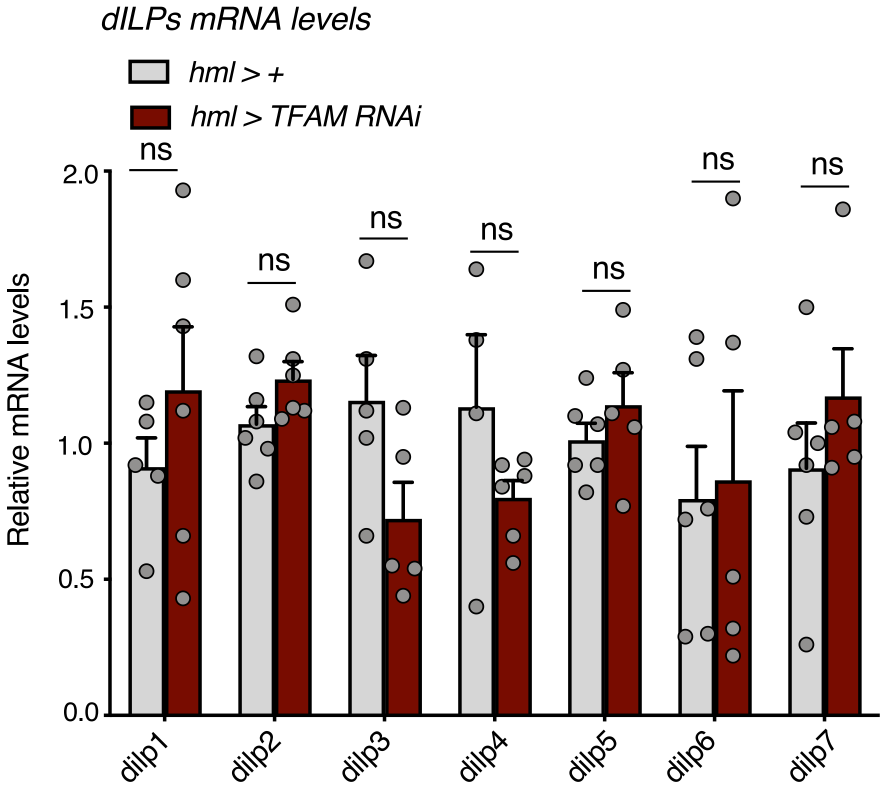

Fig. S3. Hemocyte TFAM knock down shows no change in whole larvae *diLP* mRNA levels. Whole larvae mRNA levels measured by qRT-PCR in control (*hml* > +) versus TFAM RNAi (*hml* > *UAS-TFAM-RNAi*) larvae at 96 hrs AEL. Data are represented as mean ± SEM, with individual data points plotted as symbols (*p < 0.05 and ns, not significant, unpaired t-test). n (# of samples) = 6 (control) and 6 (TFAM RNAi).

Biology Open • Supplementary information

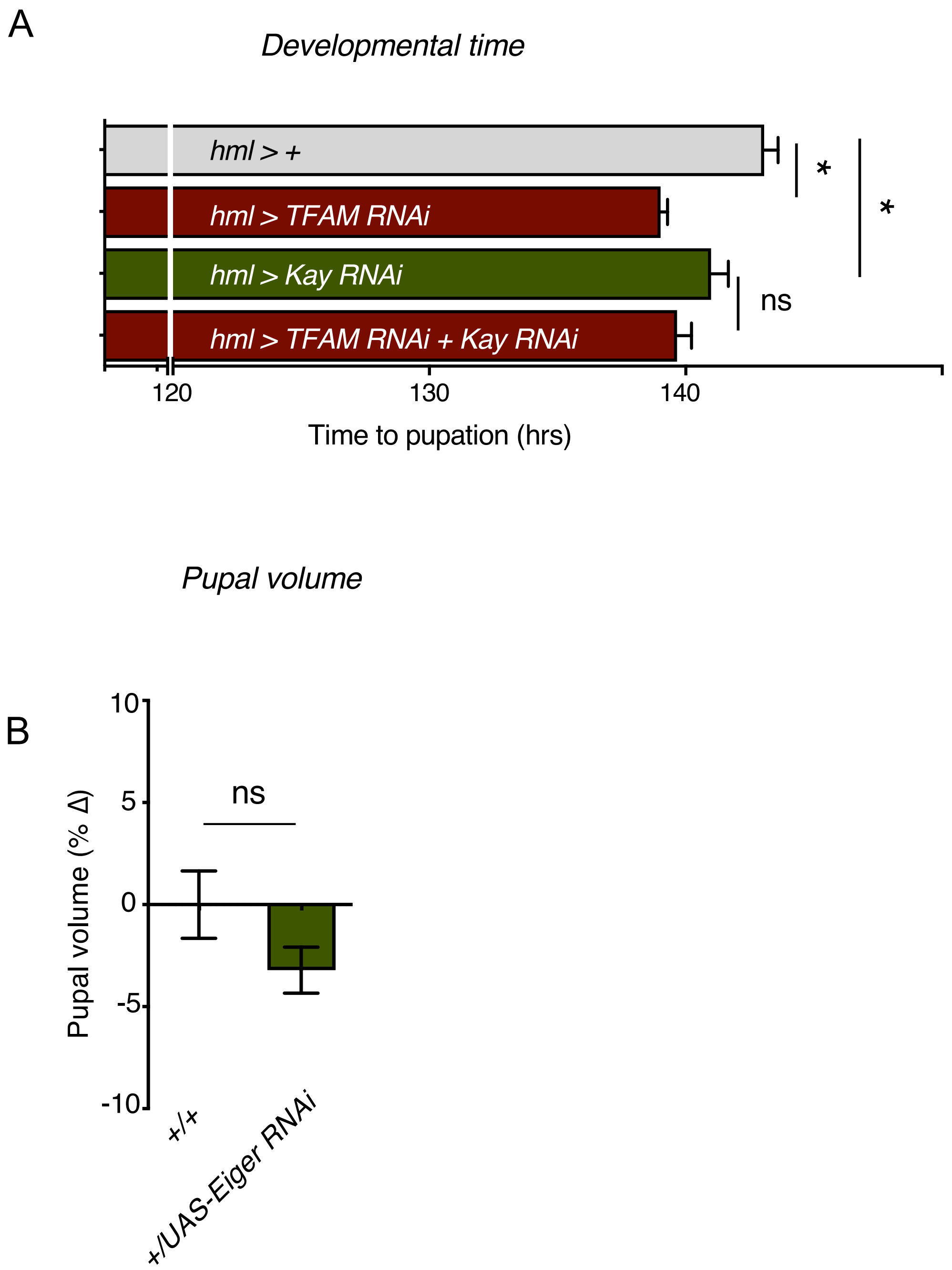

Fig. S4. Hemocyte specific knock down if JNK pathway components suppresses systemic growth (related to Fig. 5 and 6)

(**A**) Time to pupation was measured in control (*hml* > +), TFAM RNAi (*hml* > *UAS-TFAM RNAi*), Kay RNAi (*hml* > *UAS-Kay RNAi*) and TFAM RNAi + Kay RNAi (*hml* > *UAS-TFAM RNAi* + *UAS-Kay RNAi*) larvae. Data are represented as mean ± SEM, with individual data points plotted as symbols (*p < 0.05 and ns, not significant, unpaired t-test). n (# of samples) = 278 (control), 460 (*TFAM RNAi*), 128 (*Kay RNAi*), and 126 (*TFAM RNAi* + *Kay RNAi*).

(**B**) Relative change in pupal volume was calculated based on the average value of control (+/+) animals. Data are presented as mean +/- SEM (*p < 0.05, Mann-Whitney U test) for controls and Eiger RNAi lines (*+/UAS-Eiger-RNAi*). n (# of pupae) = 76 (control) vs 148 (Eiger RNAi).

Biology Open • Supplementary information

Table S1. List of primer sequences used in this study.

Name	Sequences	Reference
RpL32 (Rp49)	Fwd: GGCCCAAGATCGTGAAGAAG<br>Rev: ATTTGTGCGACAGCTTAGCATATC	Cho *et al.*, 2018
18S rRNA	Fwd: CCTGCGGCTTAATTTGACTC<br>Rev: ATGCACCACCACCCATAGAT	
4E-BP	Fwd: GCTAAGATGTCCGCTTCACC<br>Rev: CCTCCAGGAGTGGTGGAGTA	
InR	Fwd: GCTGCATCTCCTGTCGAAAT<br>Rev: CGTTGGACAGTGGGTGATAC	
TNFα/Eiger	Fwd: GATGGTCTGGATTCCATTGC<br>Rev: TAGTCTGCGCCAACATC ATC	Brandt *et al.*, 2004
dILP1	Fwd: ACAACGGTGCAGCAGTACAT<br>Rev: CCTTGGCAGCGTATTAAAGC	Cho *et al.*, 2018
dILP2	Fwd: ATGGTGTGCGAGGAGTATAATCC<br>Rev: TCGGCACCGGGCATG	
dILP3	Fwd: AGAGAACTTTGGACCCCGTGA A<br>Rev: TGAACCGAACTATCACTCAACAGTCT	
dILP4	Fwd: GCGGAGCAGTCGTCTAAGGA<br>Rev: TCATCCGGCTGCTGTAGCTT	
dILP5	Fwd: GAGGCACCTTGGGCCTATTC<br>Rev: CATGTGGTGAGATTCGGAGCTA	
dILP6	Fwd: CGATGTATTTCCCAACAGTTTCG<br>Rev: AAATCGGTTACGTTCTGCAAGTC	
dILP7	Fwd: CAAAAAGAGGACGGGCAATG<br>Rev: GCCATCAGGTTCCGTGGTT	
TFAM	Fwd: TGCAACAAGTTCCCCGTGAT<br>Rev: GCTAGGGGCCTGACTTTGTT	Zhang *et al.*, 2015
mt:ATPase6	Fwd: TGGATGAATTAATCATACACAACAT<br>Rev: AGGTATAAGAATAGCGGGTGTTC	
mt:CoIII	Fwd: CAGACTCAATTTATGGATCAACATT<br>Rev: AAAGTTGTTCCGATTAATACATGAA	

Biology Open • Supplementary information
